# Bayesian analysis of trial-based cost-effectiveness in the presence of missing data: effects of including covariates on efficiency of estimation in the ASTER trial

**DOI:** 10.1186/1745-6215-12-S1-A43

**Published:** 2011-12-13

**Authors:** Linda Sharples, Christopher Jackson, Ella Wheaton, Robert Rintoul

**Affiliations:** 1 MRC Biostatistics Unit, Cambridge, UK; 2 Cancer Research UK, London, UK; 3 Papworth Hospital NHS Foundation Trust, Cambridge, UK

## Objectives

Bayesian estimation of missing resource use data and expected costs in the ASTER trial of endosonographic staging followed by surgical staging if negative (ES), compared with surgical staging alone (SS), in candidates for lung cancer surgery.

To assess how covariates, that are included in the model to justify a “missing at random” assumption, affect estimates of expected costs.

## Methods

ASTER was a prospective, international, open-label, randomised-controlled study, with a trial-based economic analysis over 6 months. Due to delays in starting the health economic study, resource use data were collected prospectively for the second half of the study only. Although resource use data could be ascertained retrospectively, some items were difficult to ascertain once patients had been discharged from the trial centre. A Bayesian parametric model was developed to estimate missing resource use items and expected costs. Missing resource use data were modelled using Binomial, Poisson, over-dispersed equivalents of these, or using a hurdle count model if only a proportion of the patients had the event (e.g. chemotherapy). Covariates considered were randomisation group, centre, age, sex and stage of lung cancer. The total expected cost was calculated as the sum of the resource use component-specific expected costs for each randomisation group.

## Results

ES was more sensitive, resulted in fewer futile thoracotomies and had better utility during staging than SS. All patients had initial diagnostic tests and management recorded but subsequent resource use components were missing for 10-20% of cases, and only 71% had complete resource use data. Using the complete cases only, the mean 6 month cost of ES was £10,614 (£8515, £13,073) per patient versus £11,788 (£9053, £15,321) for SS, mean difference £1174 (-£948, £3912), so that ES was cheaper but with considerable uncertainty in these estimates. The Bayesian model aimed to recapture power lost due to missing data and when randomisation group was the only covariate, point estimates were reduced by 5% and posterior standard deviations were reduced by 10% compared with complete case analysis. Inclusion of other covariates resulted in small subgroups, imprecise point estimates for covariates and a resulting increase in the posterior variance for expected costs.

## Conclusions

Resource use in patient groups is highly variable and trials are rarely powered for secondary outcomes that drive costs, so that inclusion of many parameters in a Bayesian analysis may result in inefficient estimation. Covariate selection should consider both the missing data mechanism and efficiency.

